# Anti-Her2 CAR-NK92 Cells and Their Exosomes: Generation, Characterization, and Selective Cytotoxicity Against Her2-Positive Tumor Cells

**DOI:** 10.3390/ijms26157648

**Published:** 2025-08-07

**Authors:** Alexandru Tîrziu, Florina Maria Bojin, Oana Isabella Gavriliuc, Roxana Maria Buzan, Lauriana Eunice Zbîrcea, Manuela Grijincu, Virgil Păunescu

**Affiliations:** 1 Department of Functional Sciences, “Victor Babes” University of Medicine and Pharmacy, Tudor Vladimirescu Street, No. 14, 300174 Timisoara, Romania; alexandru.tirziu@umft.ro (A.T.); florinabojin@umft.ro (F.M.B.); vpaunescu@umft.ro (V.P.); 2 Center for Gene and Cellular Therapies in the Treatment of Cancer Timisoara-OncoGen, Clinical Emergency County Hospital “Pius Brinzeu” Timisoara, No. 156 Liviu Rebreanu, 300723 Timisoara, Romania; buzan.roxana@umft.ro (R.M.B.); zbircea.lauriana@umft.ro (L.E.Z.); grijincu.manuela@umft.ro (M.G.); 3 Immuno-Physiology and Biotechnologies Center, Department of Functional Sciences, “Victor Babes” University of Medicine and Pharmacy, No. 2 Eftimie Murgu Square, 300041 Timisoara, Romania

**Keywords:** chimeric antigen receptor, NK92 cells, CAR-NK cells, exosomes, Her2, immunotherapy, cytotoxicity

## Abstract

Chimeric antigen receptor (CAR)-engineered NK cells are a promising approach for targeted immunotherapy in Her2-positive cancers. This study aimed to generate anti-Her2 CAR-NK92 cells, to evaluate their selective cytotoxicity against Her2-positive cancer cells, and to isolate and characterize their released exosomes. NK92 cells were electroporated with piggyBac transposon vectors encoding anti-Her2 CAR and the helper transposase. Puromycin selection was performed to enrich the transduced cells. CAR and GFP expression were assessed by flow cytometry, and exosomes were isolated and characterized in terms of protein cargo and surface protein expression. Cytotoxicity was evaluated using real-time cell analysis against Her2-positive SK-BR3 cells and Her2-negative MCF-7 cells. Electroporation did not significantly affect NK92 cell viability. Puromycin selection efficiently enriched for CAR-expressing cells, with GFP positivity reaching 99.8% and a 15-fold increase in CAR surface expression compared to wild-type cells. CAR-NK92 cells demonstrated robust, Her2-specific cytotoxicity in a E:T-dependent manner, with the greatest effect observed at a 10:1 effector-to-target ratio. Exosomes derived from CAR-NK92 cells contained CAR molecules and selectively targeted Her2-positive cells. Anti-Her2 CAR-NK92 cells and their exosomes exhibit potent and selective cytotoxicity against Her2-positive cancer cells, supporting their potential as innovative immunotherapeutic agents for solid tumors.

## 1. Introduction

Chimeric antigen receptor (CAR)-engineered immune cell therapies have revolutionized the landscape of cancer treatment, offering highly specific and potent cytotoxicity against hematologic malignancies. However, their application in solid tumors has been limited by challenges such as tumor antigen heterogeneity, immunosuppressive tumor microenvironment, and significant off-tumor toxicities [1,2]. Natural killer (NK) cells are key effectors of the innate immune system that present several advantages over T cells, including the ability to recognize and eliminate malignant cells independent of antigen presentation and a lower risk of graft-versus-host disease (GvHD) [3]. The absence of T cell receptor molecules (TCR) make them suitable candidates for “off-the-shelf” immunotherapies against various types of cancer [4]. Recent advances in genetic engineering have enabled the development of CAR-NK cells, which combine the natural cytotoxicity and safety profile of NK cells with the targeted specificity of CAR constructs.

Although proven effective in hematological malignancies, when applied to solid tumors, CAR-bearing cell-based therapies may exhibit several limitations imposed by the tumor microenvironment and by the risk of potential toxicities. The tumor microenvironment poses a significant hurdle, especially in solid tumors, due to its immunosuppressive nature (low pH, hypoxia, overexpression of immunosuppresive cells and molecules), physical barriers generated by tumor-associated fibroblasts, and the heterogeneity of tumor cells [5]. This complexity often results in inadequate CAR-T cell infiltration, reduced expansion, and compromised persistence, thereby limiting the overall therapeutic efficacy [6].

CAR-NK cells present a promising alternative with a potentially superior safety profile and broader applicability [7]. CAR-NK cells express a lower risk of graft-versus-host disease (GVHD), enabling allogeneic “off-the-shelf” use without HLA-TCR mismatch [8]. NK cells possess inherent mechanisms for distinguishing and eliminating abnormal cells while preserving healthy tissues, mediated by a complex interplay of activating and inhibitory receptors [9]. CAR-NK cells also offer the possibility of utilizing allogeneic sources, which can streamline manufacturing processes and reduce the cost and time associated with patient-specific CAR-T cell production [10].

The field of CAR-NK-based therapies has gained interest in recent years as a promising approach for cancer immunotherapy. Many preclinical studies targeting Her2+ tumor cells in glioblastoma, rhabdomyosarcoma, and breast cancer provide evidence of their potent cytotoxic effects and lower toxicity profiles. Notably, CAR-NK cells have demonstrated greater antitumor efficacy against the BT-474, SK-BR3, and MDA-MB453 breast cancer cell lines compared to their CAR-T counterparts [11]. However, most studies have focused on lentiviral transduction rather than exploring alternative engineering strategies.

Clinically, there are currently 109 clinical studies investigating CAR-NK cell therapy against both hematological and solid tumors (clinicaltrials.gov, accessed on 25 July 2025), mostly in phase 1 or 2. Prior studies on CAR-NK cell therapies have demonstrated that this novel approach exhibits high tolerability and lower toxicity relative to CAR-T cells, along with significant potential for achieving complete remission in hematological malignancies [12].

Chimeric antigen receptors are fusion proteins that comprise an extracellular domain (usually derived from the scFv of monoclonal antibodies) that recognize a tumor-associated antigen linked to a transmembrane domain and an intracellular domain [13]. CAR engineering provides a tumor-targeted immune response independent of antigen-presentation as well as co-stimulatory molecule activation with subsequent effector cell expansion and cytokine production [14]. First-generation CARs were produced by fusing the scFv extracellular domain to a transmembrane domain and the CD3ζ molecule to allow intracellular signaling. However, their persistence and activation in vivo were proven ineffective. To overcome this limitation, second and third-generation CARs were generated by introducing co-stimulatory sequences derived from 4 to 1BB, OX40 and ICOS [15,16] to improve their persistence and stability in vivo [17,18]. To generate CAR-bearing immune effector cells, a gene encoding the CAR molecule must be introduced into the cells, which will be translated into the CAR protein [19].

Cell transfection involves the transfer of an exogenous genetic material into target cells to express a specific protein encoded by the introduced genetic material. Various methods of cell transfection have been studied on NK cells, each of them possessing specific advantages and limitations. Lentiviral transduction, which was proven very effective in CAR-T cell production, has an efficiency of less than 15% in NK cells [20]. The poor transduction efficiency of NK cells is attributed to a low expression of LDL-receptor, which is the target molecule for VSV-G lentiviruses, as well as the innate anti-viral response molecules which recognize the exogenous genetic material and cleaves it [21]. To improve the transduction efficiency, spinoculation can be employed by centrifuging the NK cell-lipid particles at 1800× *g* for 1 h to improve the contact between the cells and the particles. However, centrifugation at high forces damages the cells and reduces their viability [22]. Lipofection is another transduction strategy in which lipid-coated genetic material is incubated with the target cells so that the membranes will fuse and the genetic material would be transferred into the NK cytoplasm. Lipofectamine, a common reagent for co-transfection, contains lipid groups that spontaneously form liposomes in aqueous environments, effectively capturing the charge of delivered nucleic acids [23]. Cell nucleofection involves the transfer of genetic material by creating pores in the cell membrane through which the nucleic acid can enter in the cytoplasm. However, if mRNAs are used, the expression of the desired molecule is transient, requiring multiple rounds of nucleofection to produce CAR-bearing cells [24,25].

Transposons are mobile genetic elements that integrate into the genetic material either through a copy-and-paste, or through a cut-and-paste mechanism in the inverse tandem repeats (ITR) regions of DNA. To improve genetic material integration, a helper enzyme called a transposase is simultaneously transfected into the cells, which generates nicks in the cellular DNA, allowing seamless integration. Compared to other transposases such as Tol2 and Sleeping Beauty, the piggyBac transposase exhibits the highest transposition activity in mammalian cells, retaining activity even with minimal transposase amounts [26].

Exosomes derived from transfected cells may overcome some CAR cell therapy limitations, bearing the potential for an “off-the-shelf” therapy.

Exosomes are nanosized vesicles secreted by nearly all cell types that have emerged as promising candidates for drug delivery due to their inherent biocompatibility, ability to cross biological barriers (including the blood–brain barrier and the blood-tumor barrier), and potential for targeted delivery [27]. Acting as mediators of intercellular communication, these vesicles transport a variety of biomolecules, including proteins, lipids, and nucleic acids, influencing the behavior and function of recipient cells [28].

Exosomes interact with target cells through several distinct and complementary mechanisms. First, exosomal surface proteins can directly engage with specific receptors on the target cell membrane, triggering downstream signaling pathways via protein–protein interactions [29]. Second, exosomal membrane proteins may be enzymatically cleaved by extracellular proteases, unveiling new epitopes or binding domains that facilitate additional interactions with cell surface receptors. Third, exosomes are capable of fusing directly with the plasma membrane of the target cell, a process that enables the direct delivery of both membrane proteins as well as their cargo, including microRNAs (miRNA), messenger RNAs (mRNA), DNA, and proteins into the cytoplasm, thereby modulating gene expression and cellular function [30]. Lastly, exosomes can be internalized by recipient cells through endocytic pathways such as clathrin-mediated endocytosis, caveolin-dependent uptake, or phagocytosis [31].

While numerous studies have investigated the cytotoxic potential of NK-derived exosomes and their utility as drug delivery vehicles, research specifically focusing on exosomes derived from CAR-NK cells remains limited. A notable study by Tao et al. investigated CAR-NK cell-derived exosomes loaded with a transferrin receptor-binding peptide (T7) and mPEG-TK-Ce6@RSL3, a reactive oxygen species (ROS)-generating compound. These engineered exosomes demonstrated the ability to cross the blood–brain barrier and exhibited selective antitumor activity mediated by the CAR on their surface against Her2-positive breast cancer cells in a murine model [32].

Given the limitations of NK cell transduction, this study aims to generate and characterize anti-Her2 CAR-NK92 cells using transposon-based genetic engineering. Moreover, given the potential of exosomes as “off-the-shelf” cell-free therapy to influence various cellular processes, along with their physical properties that allow crossing the blood-tumor barriers [1,33,34], another aim of this study is to characterize exosomes released from the engineered CAR-NK cells.

## 2. Results

### 2.1. Plasmid Electroporation Does Not Significantly Affect NK92 Cell Viability

**Figure 1:**
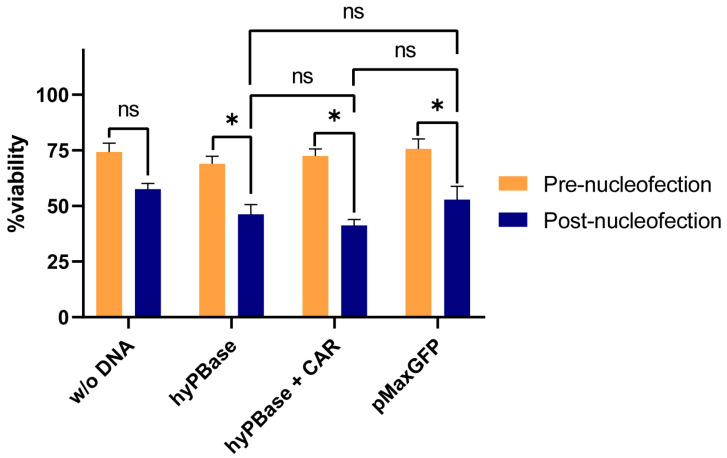
NK92 cell viability before and after nucleofection across different experimental conditions. Bar chart showing the percentage of viable cells pre-nucleofection (orange) and post-nucleofection (blue) for each condition: w/o DNA, hyPBase, hyPBase + CAR, and pMaxGFP. Data are presented as mean ± SEM. Statistical significance was determined using paired *t*-tests; * *p* < 0.05, ns = not significant. Nucleofection significantly reduced cell viability in the hyPBase, hyPBase + CAR, and pMaxGFP groups, but not in the w/o DNA group. No significant differences in post-nucleofection viability were observed among the different plasmid conditions.

NK92 cells were electroporated in four conditions—without any plasmid, with hyPBase, hyPBase and CAR, and pmaxGFP. Cells nucleofected without DNA exhibited a mild decrease in viability after electroporation, without statistically significant differences (*p* = 0.2377, t = 2.553, df = 1). However, the plasmid electroporation decreased cell viability significantly in all three groups (hyPBase − *p* = 0.0306, t = 20.79, df = 1; hyPBase + CAR − *p* = 0.01, t = 57.72, df = 1; pMaxGFP − *p* = 0.0399, t = 15.94, df = 1). Compared to the populations transfected with a single plasmid (hyPBase, pmaxGFP), NK92 cells nucleofected with both hyPBase and CAR-PB vector manifested a higher decrease in cell number. The change in cell number at 1 day post-nucleofection between groups was not statistically significant (hyPBase vs. hyPBase + CAR − *p* = 0.22, t = 2.77, df = 1; hyPBase vs. pMaxGFP − *p* = 0.1403, t = 4.465, df = 1; hyPBase + CAR vs. pMaxGFP − *p* = 0.1751, t = 3.543, df = 1) (Figure 1).

### 2.2. Puromycin Toxicity Curves

At the control condition (no puromycin), the untransduced NK92 cells exhibit normal cell proliferation over time, with cell numbers increasing from approximately 3 × 10^5^ cells at day 0 to 1.3 × 10^6^ cells by day 3, and further increasing to about 1.5 × 10^6^ cells by day 7.

**Figure 2:**
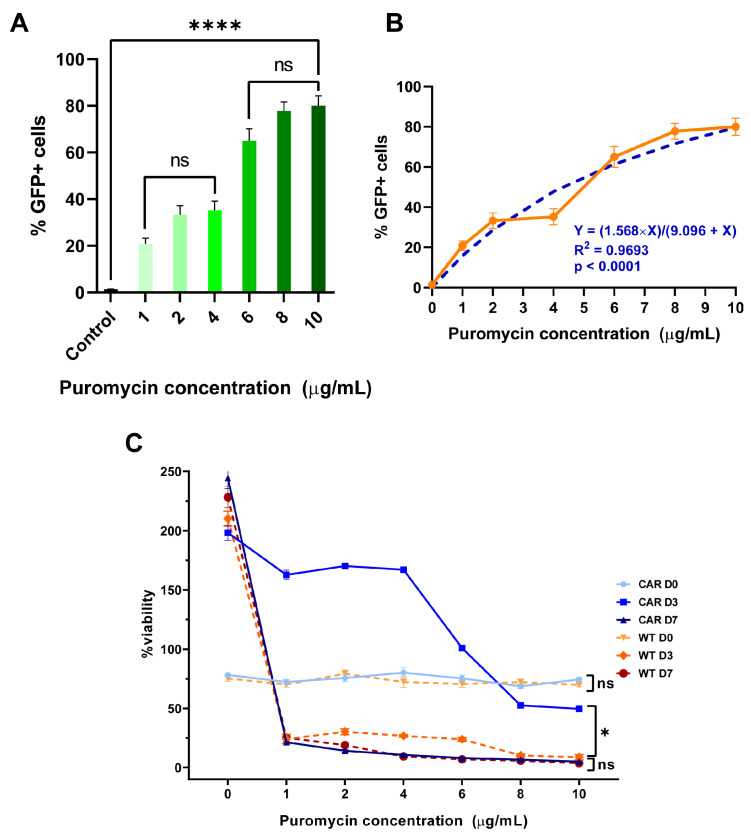
(**A**) Effect of Puromycin Concentration on GFP+ Cell Percentage. Bar graph showing the percentage of GFP-positive cells after selection with increasing concentrations of puromycin (1–10 μg/mL) compared to an untreated control. A significant increase in GFP+ cells was observed with puromycin treatment compared to control (****, *p* < 0.0001). No significant differences (*ns*) were found among the 1–4 μg/mL group or between 6 and 10 μg/mL, indicating a plateau in selection efficiency at higher concentrations. Error bars represent standard error of the mean (SEM). (**B**) Dose-dependent effect of puromycin on GFP-positive cell percentage at day 3 of selection. Data points (orange curve) represent mean ± SEM. The blue dashed line shows a nonlinear regression fit (Y = (1.568 × X)/(9.096 + X)), with R^2^ = 0.9693 and *p* < 0.0001, indicating a strong and statistically significant fit. The response displays a saturation curve, with maximal GFP+ cell percentage reached at higher puromycin concentrations. (**C**) Viability of CAR-transduced (CAR) and wild-type (WT) cells at days 0, 3, and 7 post-transduction following treatment with increasing concentrations of puromycin. CAR D3 cells (blue squares) show significantly higher viability at intermediate puromycin concentrations compared to other groups (* *p* = 0.0102, t = 3.538, df = 6). No significant differences (ns) were observed among other groups at D0 or D7 (D0: *p* = 0.2398, t = 1.305, df = 6; D7: *p* = 0.5294, t = 0.6673, df = 6), suggesting both a dose-dependent and a time-dependent toxicity.

As puromycin concentration increases, several patterns emerge, a dose-dependent growth inhibition was observed on short-term exposure (from day 0 to day 3). At lower concentrations (1–4 μg/mL), moderate growth inhibition compared to control was observed. In contrast, at higher concentrations (6–10 μg/mL), a significant growth inhibition with cell numbers reduced to approximately 2 × 10^5^ was observed. After 7 days of puromycin exposure, NK92 cell experience severe toxicity even at the lowest concentration (1 μg/mL), as the cell numbers drop dramatically compared to control. At concentrations ≥ 2 μg/mL, cell numbers are reduced significantly, indicating profound cytotoxicity (Figure 2C).

The NK92 transduced cells show healthy proliferation in the absence of puromycin, though at lower rates than untransduced cells, increasing from ~3 × 10^5^ cells (day 0) to ~4.2 × 10^5^ (day 3) and ~7.5 × 10^5^ (day 7). Day 3 counts show moderate decline at all concentrations (~10^5^ at 1–4 μg/mL decreasing to ~3–4 × 10^4^ at 8–10 μg/mL). However, at day 7, cells demonstrate selective resistance, as at a puromycin concentration of 1–4 μg/mL cells maintain viability (~10^5^ and ~7 × 10^4^ cells, respectively). In contrast, at ≥4 μg/mL, progressive decline with minimal survival at higher concentrations was observed (Figure 2C).

**Figure 3:**
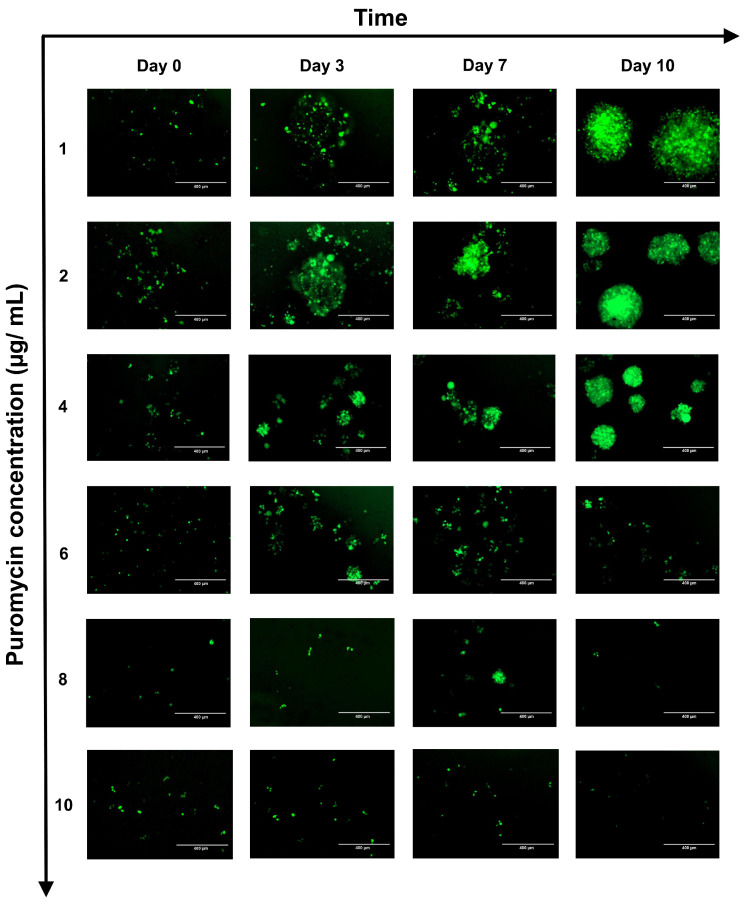
Selection of NK92 cell niches resistant to puromycin due to the *pac* gene. A dose-dependent effect is observed, accelerating the selection of resistant niches up to a concentration of 4 μg/mL of puromycin. Once this dose is exceeded, a decline in GFP-positive cells is observed—a dose-dependent effect, which overcomes the activity of puromycin-acetyl transferase.

At Day 3, the percentage of GFP-positive cells increases with increasing puromycin concentrations (*p* < 0.0001, F = 64.21). At lower concentrations (1–4 μg/mL) the percentage of GFP+ cells increases compared to control, but without statistical significance (*p* = 0.1125, F = 4.935) whereas at higher concentrations (6-10 μg/mL), the percentage of GFP-positive cells is high and relatively stable (*p* = 0.1808, F = 3.192). At day 7 of selection, the percentage of GFP-positive cells treated with lower puromycin concentrations (1–4 μg/mL) increases but remains similar or slightly decreases at higher concentrations (6–10 μg/mL), suggesting that the higher puromycin concentrations overcome the puromycin resistance mechanisms (Figure 2A,B and Figure 3).

### 2.3. Transfected Cells Express GFP and CAR Molecules

**Figure 4:**
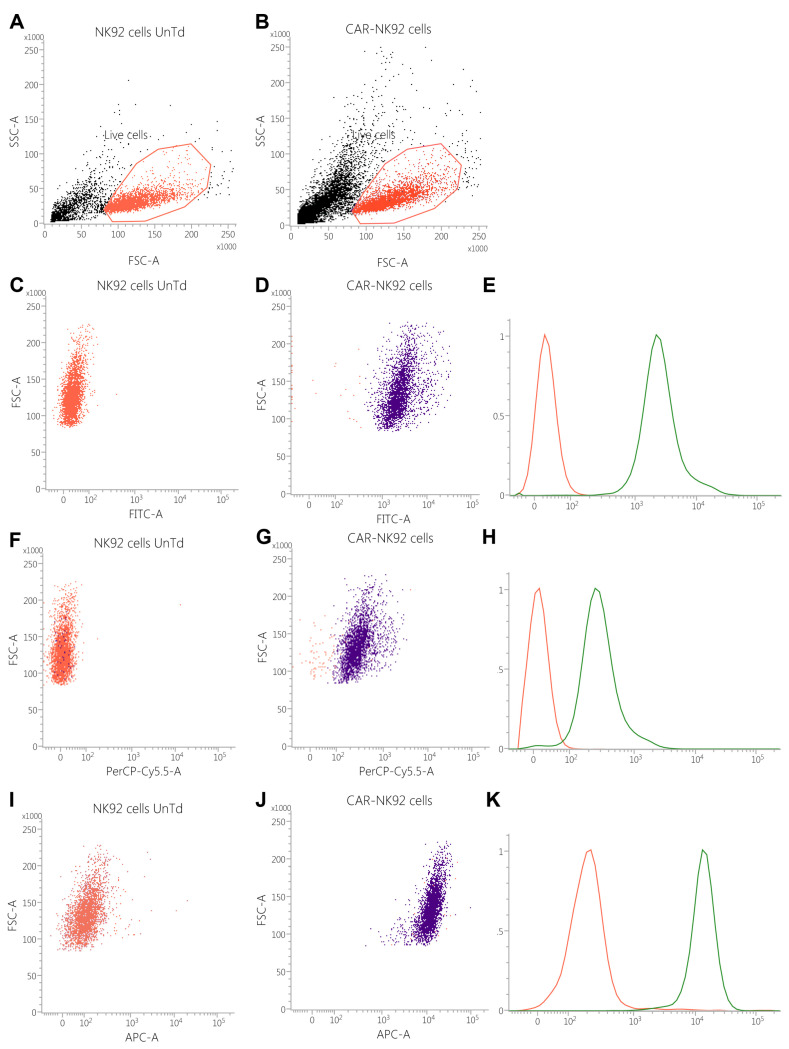
Comparative flow cytometric analysis between untransduced and transduced NK92 cells. (**A**,**B**). Gating strategy for identifying live cells (red polygon). (**C**,**D**) GFP expression in transduced cells is significantly higher compared to untransduced cells, suggestive for successful transfection. (**E**) Fluorescence intensity histograms showing significant differences between the two groups (37.33 ± 4.13 vs. 3395.66 ± 111.065 MFUs, *p* < 0.001, df = 4, t = 55.5200). (**F**,**G**). CAR expression analysis using anti-G4S linker antibodies revealed a significantly higher expression of G4S linker (from the CAR molecule) in the transduced cell population. (**H**) Fluorescence intensity histogram showing a statistically significant difference in fluorescence emission in the PerCP spectrum in the transduced cell population (32.67 ± 28.41 MFUs vs. 486 ± 63.16 MFUs, *p* = 0.002, df = 4, t = 7.0600). (**I**,**J**). Anti-kappa light chain expression was significantly higher in the transduced cell population, with statistically significant differences as observed in the histogram ((**K**), 13,355.5 ± 832.958 MFUs vs. 5473.5 ± 144.828 MFUs, *p* = 0.0045, df = 4, t = 14.9193).

Based on the linear FSC vs. SSC dot-plot, untransduced NK92 cells expressed a cell viability of 75.83% compared to 68.53% in the transduced population, without statistically significant differences (*p* = 0.855). From the live cell populations, the GFP positive cells were selected on the logarithmic FITC scale, which has shown that 0.24% of untransduced cells were GFP+ compared to 99.81% in the transduced population (*p* = 0.0260). Based on the median fluorescence intensity, the MFI of NK92 WT was 37.33 ± 4.13 vs. 3395.66 ± 111.065 MFUs in the transduced cell population (*p* < 0.001, df = 4, t = 55.5200) (Figure 4C–E).

After incubating the cells with primary mAb against the G4 S linker, the median fluorescence intensity revealed a 15-fold difference in fluorescence between the two populations, as NK92 WT cells expressed 32.67 ± 28.41 MFUs compared to 486 ± 63.16 MFUs in the electroporated NK92 population (*p* = 0.002, df = 4, t = 7.0600) (Figure 4F–H).

Anti-kappa light chain antibody binding has revealed a statistically significant difference in kappa light-chain expression in the NK92 electroporated population compared to NK92 WT (13355.5 ± 832.958 MFUs vs. 5473.5 ± 144.828 MFUs, *p* = 0.0045, df = 4, t = 14.9193) (Figure 4I–K).

### 2.4. CAR-NK92 Exert Her2-Specific Cytotoxic Effects

**Figure 5:**
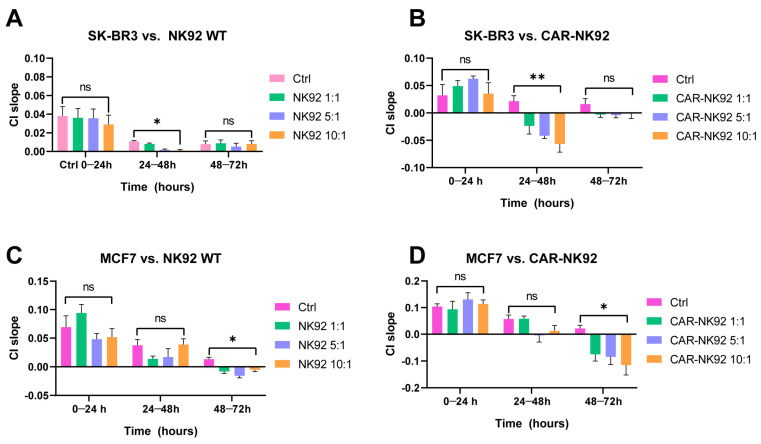
Real-time cytotoxicity analysis of NK92 and CAR-NK92 cells against breast cancer cell lines SK-BR3 and MCF7. Cell index (CI) slope was measured over three time intervals (0–24 h, 24–48 h, and 48–72 h) using xCELLigence assays. (**A**,**B**) SK-BR3 cells were co-cultured with wild-type NK92 (**A**) or CAR-NK92 (**B**) cells at different effector-to-target (E:T) ratios (1:1, 5:1, 10:1). (**C**,**D**) MCF7 cells were co-cultured with wild-type NK92 (**C**) or CAR-NK92 (**D**) cells at the same E:T ratios. Controls (Ctrl) represent target cells without effectors. Data are presented as mean ± SEM. Statistical significance was determined by appropriate tests; ns = not significant, * *p* < 0.05, ** *p* < 0.01. CAR-NK92 cells show enhanced cytotoxicity against SK-BR3 and MCF7 cells compared to wild-type NK92, particularly at higher E:T ratios and later time points.

SK-BR3 cells were seeded on a 16-well RTCA plate and after 24 h of incubation NK92/CAR-NK92 cells at different effector-to-target ratios (E:T, 1:1, 5:1, 10:1) were added. In the NK92-treated population all four groups had a positive cell index (CI) slope at 0–24 h, indicative for cell proliferation, with no statistically significant differences (F (3, 4) = 0.351, *p* = 0.8216). Between 24 and 48 h, the SK-BR3 cells from all 4 groups exhibited a decrease in proliferation rate (suggested by the decrease in cell index slope, F (3, 4) = 46.38, *p* = 0.015). However, an E:T-dependent CI-decrease was observed, suggesting that NK92 WT cells can suppress SK-BR3 cell growth in a E:T-dependent manner. Between 48 and 72 h, the SK-BR3 cells started proliferating again, which is indicative of the fact that the mechanisms for non-specific cell lysis of NK92 WT cells were overcome by the target cells (F (3, 4) = 0.401, *p* = 0.7602) (Figure 5A).

In the CAR-NK92-treated group the SK-BR3 exhibited a positive cell index slope, indicative for cell proliferation between 0 and 24 h (F (3, 4) = 1.686, *p* = 0.3086). After the addition of CAR-NK92 cells (24 h), an E:T-dependent decrease in cell number was observed, with the most negative CI slope being in the CAR-NK92 10:1 arm, indicating active cytotoxicity rather than just growth inhibition mediated by the CAR-NK92 cells (F (3, 4) = 15.93, *p* = 0.01). Between 48 and 72 h, the cell index slope remained close to 0, which suggests a persistent anti-proliferative effect of the CAR-NK92 anti-Her2 cells against the SK-BR3 cells (F (3, 4) = 2.796, *p* = 0.1729). The sustained effect of CAR-NK92 cells indicates that the Her2-targeted approach overcomes potential resistance mechanisms (Figure 5B).

Similarly, MCF-7 cells were incubated with NK92/CAR-NK92 cells at different E:T ratios (1:1, 5:1, 10:1). Between 0 and 24 h, MCF-7 cells from all groups exhibited a positive cell index slope, suggestive for cell proliferation (F (3, 4) = 4.028, *p* = 0.1059). At 24 h, the NK92 cells were added in co-culture. Between 24 and 48 h, cell proliferation rate started to decrease, with a more pronounced effect in the NK92 1:1 and NK92 5:1 treated cells (F (3, 4) = 3.128, *p* = 0.1497). Between 48 and 72 h, a negative CI slope was observed in all MCF-7 cells incubated with NK92 cells, with the most pronounced decrease in the NK92 5:1 population (F (3, 4) = 24.6, *p* = 0.049). NK92 cells express their non-specific cytotoxicity against MCF-7 cells, reflected by the negative cell index slope, suggestive of cell death (Figure 5C).

In the CAR-NK92 treated experiment MCF-7 cells exhibited a positive cell index slope, indicative for cell proliferation between 0 and 24 h (F (3, 4) = 1.085, *p* = 0.4508). Between 24 and 48 h, the cell proliferation rate decreased, with a more pronounced effect at higher E:T ratios (5:1, 10:1, F (3, 4) = 5.920, *p* = 0.06). At 48–72 h, an E:T-dependent cellular death was observed (F (3, 4) = 9.312, *p* = 0.0282). The CAR-NK92 cells started exerting their cytotoxic effects between 24 and 48 h, eventually leading to cell death, as indicated by the negative cell index slope by 48–72 h. Absence of a specific target may explain the slower onset and more variable cytotoxic effects compared to CAR-NK cell experiments (Figure 5D).

### 2.5. CAR-NK92-Derived Exosome Characterization

**Figure 6:**
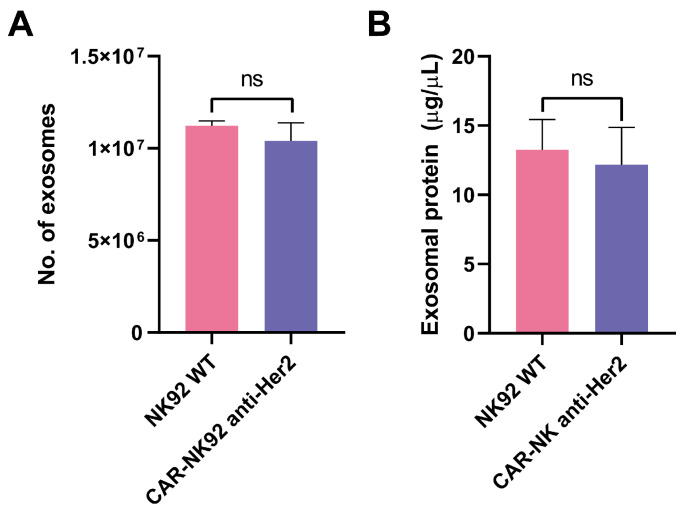
Comparison of exosome production and protein content between NK92 WT and CAR-NK92 cells. (**A**) Quantification of exosomes produced by NK92 WT and CAR-NK92 anti-Her2 cells after 72 h of incubation in serum-free media. Starting from 4 × 10^6^ cells, NK92 WT produced 11.2 × 10^6^ exosomes, while CAR-NK92 produced 10.4 × 10^6^ exosomes, with no statistically significant difference between the two groups (*p* = 0.50, n = 3). (**B**) Measurement of exosomal protein concentration showed 13.26 μg/μL for NK92 WT and 12.17 μg/μL for CAR-NK92 cells, also without significant difference (*p* = 0.78, n = 3). Data represent mean ± SEM; ns = not significant.

For 72 h, 4 × 10^6^ NK92 cells (both WT and untransduced) were incubated in serum-free media, producing 11.2 × 10^6^ exosomes (NK92 WT) and 10.4 × 10^6^ exosomes for CAR-NK92 cells, respectively, without statistically significant differences between the two populations (*p* = 0.50) (Figure 6A). The protein content of the exosomal suspensions was 13.26 μg/μL for NK92-WT and 12.17 μg/μL for CAR-NK92 (*p* = 0.78) (Figure 6B).

Western blot analysis was performed to characterize protein expression in whole cell lysates and exosomal fractions derived from NK92 WT and CAR-NK92 cells. In the whole cell lysates, both cell types showed expression of CD3ζ and granzyme B. Notably, CAR-NK92 cells displayed two distinct bands for CD3ζ, indicative of both endogenous CD3ζ and the CAR-associated CD3ζ fusion protein. Granzyme B was detected in both monomeric and multimeric forms in both NK cell lines.

Exosomal characterization confirmed the presence of exosome-specific markers CD63 and Alix in exosomes from both NK92 WT and CAR-NK92 cells, suggesting exosome biogenesis through ESCRT-dependent and Alix-mediated pathways. In contrast to the cell lysates, β-actin was undetectable in the exosomal fractions, supporting minimal contamination with cellular debris during isolation.

**Figure 7:**
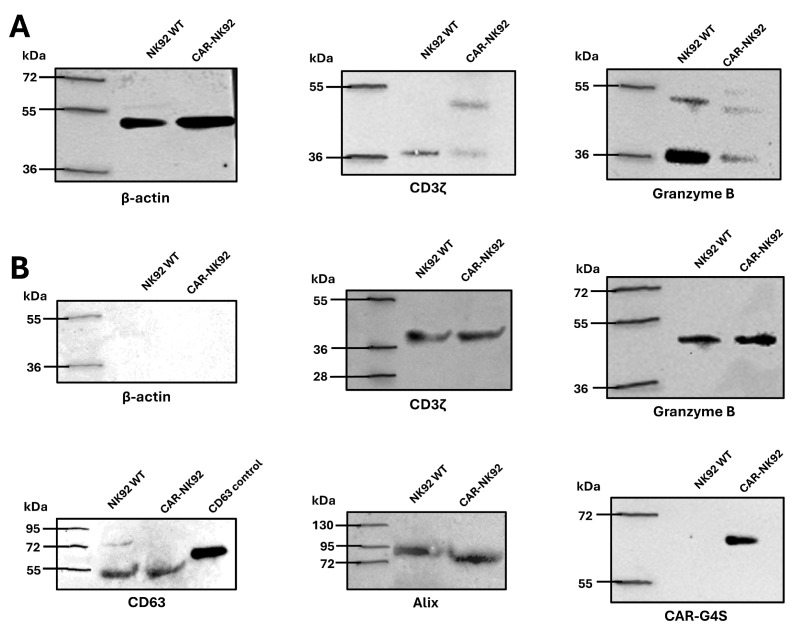
Western blot analysis of the whole cell lysate and the exosomal populations. (**A**) Both cell populations express CD3ζ, while CAR-NK92 cells express two bands containing CD3ζ, suggestive for CAR expression. Both cell populations express granzyme B, present both as monomers and multimers. Compared to whole cell lysate which stained positive for β-actin (loading control). (**B**) Exosomal lysate Western blot revealed that both exosomal populations were negative for β-actin, suggesting low contamination during isolation The exosomal specific markers CD63 and Alix were present in both exosomal populations, suggesting that exosomes are released via ESCRT-dependent and Alix-dependent pathways. Both exosomal populations express CD3ζ and granzyme B, while CAR expression, documented using the G4S antibody was present only in the exo-CAR-NK92 population; kDa—kilodaltons.

Importantly, CD3ζ and granzyme B were also detected in exosomes from both cell populations, indicating the packaging of immune effector components into extracellular vesicles. The G4S linker, used as a marker of CAR expression, was present exclusively in exosomes derived from CAR-NK92 cells, confirming selective incorporation of the CAR protein into engineered NK-derived exosomes (Figure 7).

**Figure 8:**
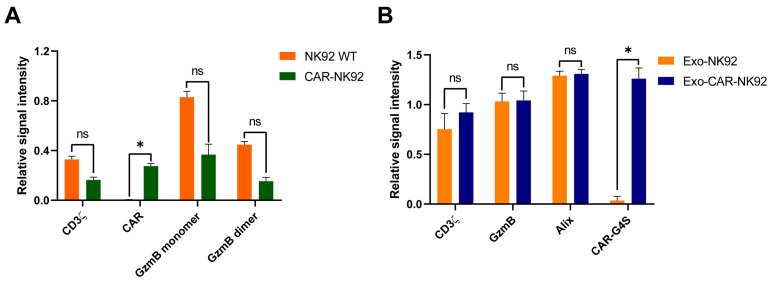
Relative protein expression levels in NK92 WT, CAR-NK92, Exo-NK92, and Exo-CAR-NK92 cells. (**A**) Bar graph showing the relative signal intensity of CD3ζ, CAR, GzmB monomer, and GzmB dimer in NK92 WT (orange) and CAR-NK92 (green) cells, normalized to β-actin as a loading control. No significant differences were observed in expression of CD3ζ (*p* = 0.1839, t = 3.365, df = 1), granzyme B monomers (*p* = 0.1753, t = 3.540, df = 1) and dimers (*p* = 0.12, t = 5.244, df = 1), while CAR expression was significantly increased in the transduced NK cell population (*p* = 0.05, t = 12.791, df = 1). (**B**) Bar graph displaying the relative signal intensity of CD3ζ, GzmB, Alix, and CAR-G4S in Exo-NK92 (orange) and Exo-CAR-NK92 (blue) exosomes, normalized to CD63 as a loading control. No statistically significant differences were observed in terms of CD3ζ (*p* = 0.1328, t = 4.725, df = 1), granzyme B (*p* = 0.9187, t = 0.1285, df = 1) or Alix expression (*p* = 0.4325, t = 1.238, df = 1), while the presence of CAR was significantly increased in the Exo-CARNK92 group (*p* = 0.046, t = 13.81, df = 1). Data are presented as mean ± SEM; * *p* < 0.05, ns = not significant.

To assess the impact of CAR nucleofection on protein expression, a comparative analysis of the relative signal intensities of CD3ζ, CAR, granzyme B (GzmB) monomer, and GzmB dimer in NK92 WT and CAR-NK92 cells was performed, with normalization to β-actin as a loading control. No significant differences were observed in the expression levels of CD3ζ (*p* = 0.1839, t = 3.365, df = 1), GzmB monomer (*p* = 0.1753, t = 3.540, df = 1), or GzmB dimer (*p* = 0.12, t = 5.244, df = 1) between the two cell types. However, CAR expression was significantly increased in the CAR-NK92 population compared to NK92 WT (*p* = 0.05, t = 12.791, df = 1) (Figure 8A).

Further analysis of the proteins isolated from Exo-NK92 and Exo-CAR-NK92 focusing on the relative signal intensities of CD3ζ, GzmB, Alix, and CAR-G4S was performed. Protein expression was normalized to CD63 as a loading control. No statistically significant differences were observed in the expression of CD3ζ (*p* = 0.1328, t = 4.725, df = 1), GzmB (*p* = 0.9187, t = 0.1285, df = 1), or Alix (*p* = 0.4325, t = 1.238, df = 1) between exosome groups. In contrast, CAR-G4S expression was significantly elevated in the Exo-CAR-NK92 group compared to Exo-NK92 (*p* = 0.046, t = 13.81, df = 1) (Figure 8B).

**Figure 9:**
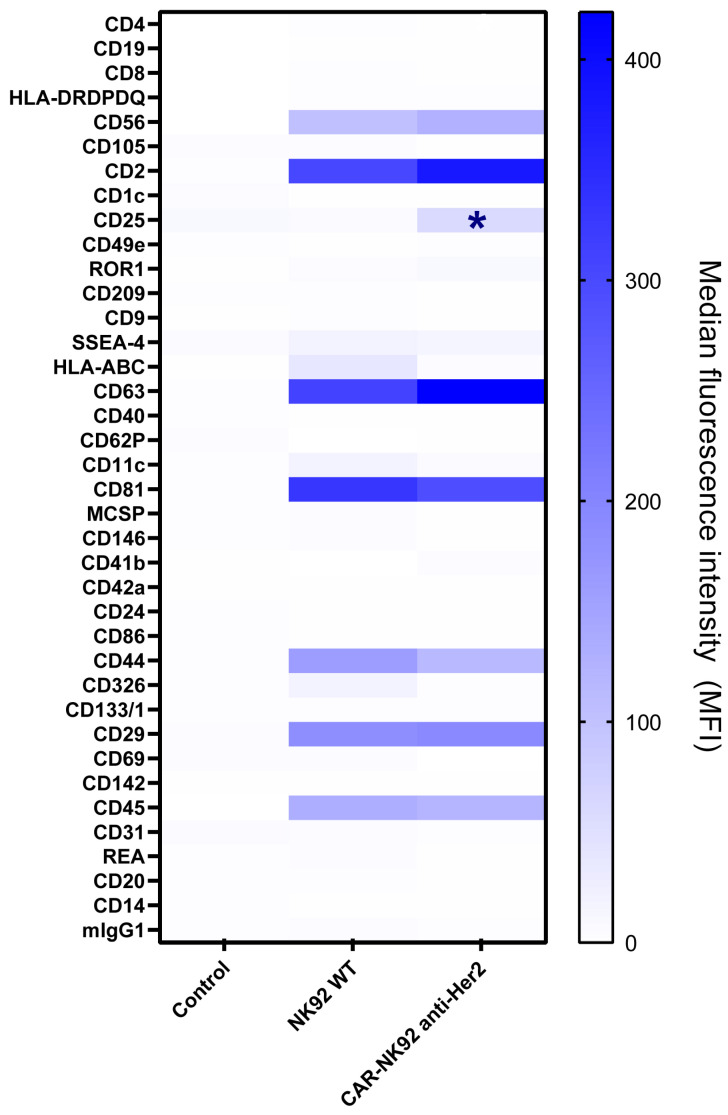
Exosomal surface protein characterization using MACSPlex revealed that both exosomal populations express the classical tetraspanins CD63 and CD81. However, CD9 was not detected. Both exosomal populations expressed CD56, an NK-cell specific marker, reflecting their origin. Additionally, both exosomal populations expressed CD2, CD44, CD29 and CD45. Exosomes from CAR-NK92 cells expressed higher levels of CD25; *—*p* < 0.05.

Comparative surface proteome analysis between Exo-NK92 and Exo-CAR-NK92 was assessed via the MACSPlex EV detection system. All data is presented as comparative analyses between Exo-NK92 and Exo-CAR-NK92, expressed in median fluorescence units (MFUs). The flow cytometric analysis has revealed that both exosomal populations express high levels of CD63, CD81, and CD2 (CD63: 311 vs. 421.5, *p* = 0.337; CD81: 332 vs. 291, *p* = 0.636; CD2: 304 vs. 381, *p* = 0.307). CD326, HLA-A/B/C, CD11c are mildly expressed in both populations, without significant differences (CD326: 19.5 vs. 16.5, *p* = 0.698; HLA-A/B/C: 39.5 vs. 4, *p* = 0.08; CD11c: 19.5 vs. 7.5, *p* = 0.275). CD29 and CD45 were upregulated in both exosomal populations (CD29: 196 vs. 185.7, *p* = 0.473; CD45: 132.5 vs. 122, *p* = 0.378), while CD25 was more represented in CAR-NK92 exosomes (14.2 vs. 64.6, *p* = 0.04) (Figure 9).

### 2.6. CAR-NK92-Exo Bind Her2 Molecules on SK-BR3 Cells

**Figure 10:**
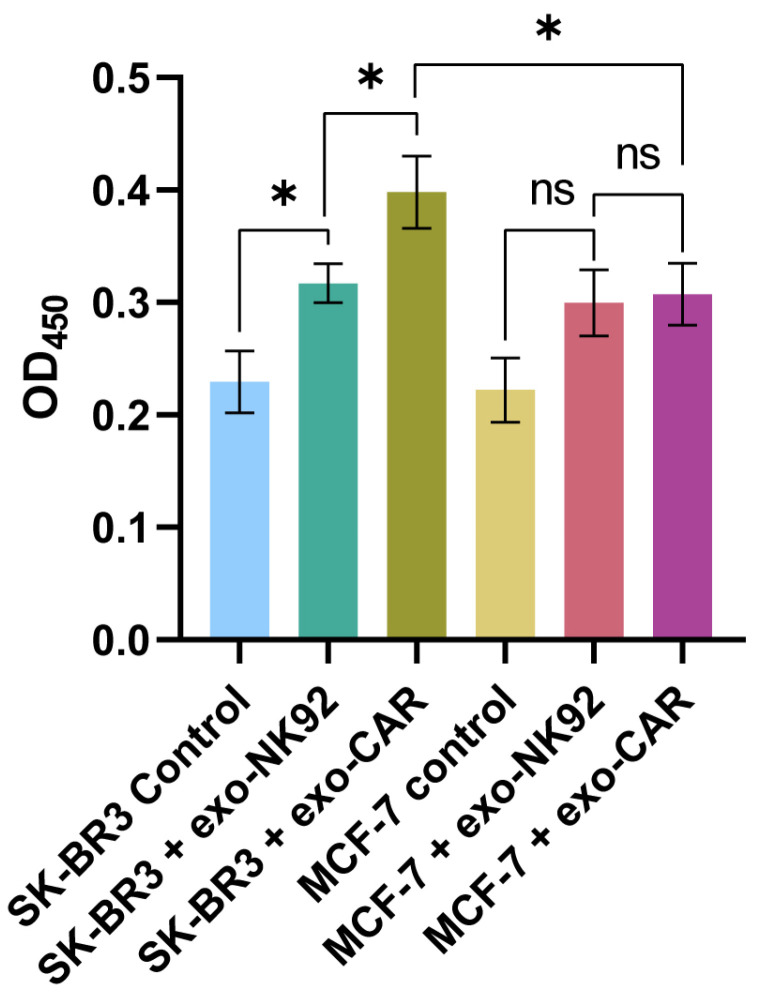
Analysis of the binding capacity of exo-NK92 and exo-CAR to SK-BR3 and MCF-7 cells using the In-cell ELISA technique. Cells were incubated with the described exosomes and subsequently stained with anti-CD63 antibodies to investigate the binding capacity of exosomes via surface markers. Absorbance intensity is directly proportional to the amount of CD63 present in the well. Control MCF-7 and SK-BR3 cells show no statistically significant differences in CD63 marker expression (*p* = 0.8562, n = 3). Positivity for this marker is associated with continuous exosome production. When the cell lines were incubated with NK92-exosomes, an increase in optical density at 450 nm was observed. The difference was significant in the SK-BR3 cell population (*p* = 0.013, n = 3) and close to statistical significance in the MCF-7 population (*p* = 0.07, n = 3). In contrast, statistically significant differences were observed in the binding of Exo-CAR-NK92 to the Her2-positive SK-BR3 cell line compared to the constitutively Her2-negative MCF-7 cell line (*p* = 0.0435, n = 3). Comparative analysis revealed that CAR incorporation did not significantly alter binding in MCF-7 cells (*p* = 0.8481, n = 3), but significantly increased exosomal binding in Her2+ SK-BR3 cells (*p* = 0.0369, n = 3); *—*p* < 0.05; ns—not significant.

SK-BR3 and MCF-7 cells were fixated and permeabilized on an ELISA plate, followed by incubation with exosomes from NK92 WT and CAR-NK92, respectively. To detect the presence of exosomes in the plate, anti-CD63 antibodies were used, conjugated with HRP. Both MCF-7 and SK-BR3 cells treated with anti-CD63 antibodies alone expressed similar absorbances at 450 nm attributed to a similar CD63 expression (SK-BR3: 0.22941 ± 0.0198 RAU vs. MCF-7: 0.2221 ± 0.0215 RAU, *p* = 0.8562). Comparative analysis of MCF-7 and SK-BR3 cells incubated with Exo-NK92 with their corresponding negative controls revealed a significant increase in the optical density at 450 nm, suggestive for exosome binding to the target cells (SK-BR3: 0.22941 ± 0.0198 RAU vs. SK-BR3-exo-NK92: 0.317041 ± 0.0124 RAU, *p* = 0.013; MCF-7: 0.2221 ± 0.0215 RAU vs. MCF-7-exo-NK92: 0.2996 ± 0.0216 RAU, *p* = 0.07). However, the difference in exosome binding between the two cell lines was not significant (0.317041 ± 0.0124 RAU vs. 0.2996 ± 0.0216 RAU, *p* = 0.6136). In contrast, when CAR-NK92 exosomes were added, the absorbance increased significantly in the SK-BR3 population compared to MCF-7, suggestive of Her2-binding capacity (SK-BR3: 0.3981 ± 0.0249 RAU vs. MCF-7: 0.3074 ± 0.0206 RAU, *p* = 0.0435). Comparative analysis between CAR-NK92-exo and their NK92 counterparts revealed statistically significant differences in the SK-BR3 population (SK-BR3-exo-NK92: 0.22941 ± 0.0198 RAU vs. SK-BR3-exo-CAR-NK92: 0.3981 ± 0.0249 RAU, *p* = 0.0369), and non-significant changes in the MCF-7 cell line (MCF-7-exo-CAR-NK92: 0.3074 ± 0.0206 RAU vs. MCF-7-exo-NK92: 0.2996 ± 0.0216 RAU, *p* = 0.8481) (Figure 10).

### 2.7. Exosomes from NK92 and CAR-NK92 Cells Exert Cytotoxic Effects

Assessment of cell viability was performed by measuring the reduction in resazurin (alamarBlue™) after 24 h of incubation with increasing concentrations of exosomes expressed as weight of exosomal protein (1, 5, and 10 µg) from either NK92-derived exosomes (exo-NK) or CAR-engineered NK92-derived exosomes (exo-CAR-NK) in SK-BR3 and MCF-7 breast cancer cell lines.

**Figure 11:**
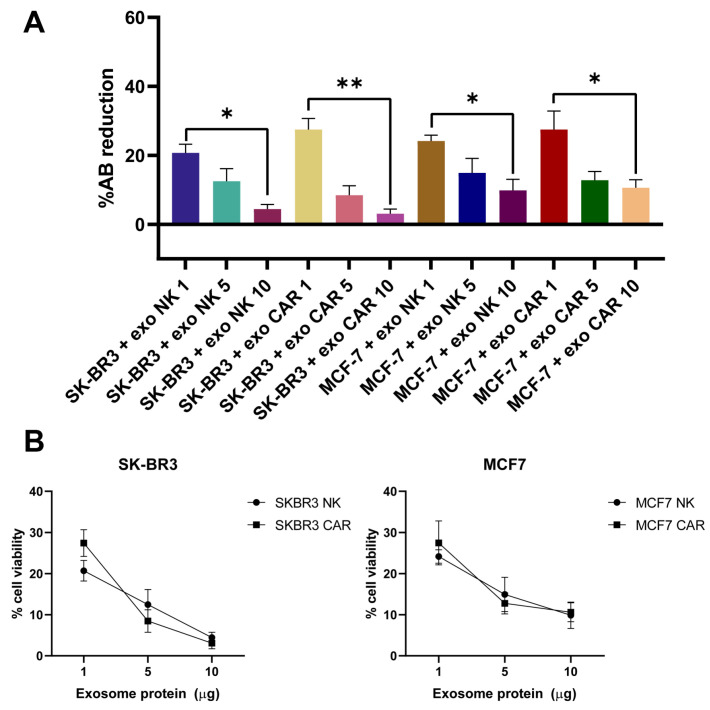
Exosome-mediated cytotoxic effects of NK and CAR-NK cells on SK-BR3 and MCF-7 breast cancer cell lines. (**A**) Percentage of resazurin (AB) reduction indicating cytotoxicity of NK cell-derived and CAR-NK cell-derived exosomes at different concentrations (1, 5, and 10 μg) against SK-BR-3 and MCF-7 breast cancer cells. While both NK and CAR-NK-derived exosomes expressed significant cytotoxic effects, the most pronounced effect was observed in SK-BR3 cells treated with 10 μg of CAR-NK-derived exosomes (*p* < 0.01). (**B**) Dose-dependent reduction in cell viability of SK-BR-3 and MCF-7 cells upon treatment with NK or CAR-NK cell-derived exosomes, as measured by viability assays. CAR-NK exosomes demonstrated greater cytotoxicity compared to NK exosomes across all tested doses. Data are presented as mean ± SEM; significance levels are denoted as * *p* < 0.05 and ** *p* < 0.01; AB—alamarBlue.

In SK-BR3 cells, treatment with exo-NK showed a dose-dependent reduction in resazurin reduction (F (2, 6) = 9.254, *p* = 0.0149). Similarily, exo-CAR-NK treatment resulted in a dose-dependent reduction in SK-BR3 viability, with a more pronounced effect at doses of 5 µg and 10 µg of exosomal protein (F (2, 6) = 24.62, *p* = 0.0013). For the MCF-7 cell line, both exo-NK and exo-CAR expressed similar reductions in cell viability (F (2, 6) = 5.187, *p* = 0.049; F (2, 6) = 6.226, *p* = 0.0344) (Figure 11).

Exosomes from CAR-NK92 cells exhibited a significantly lower IC_50_ against SK-BR3 cells (22.361 µg/mL protein) compared to NK92 exosomes (49.919 µg/mL protein), indicating enhanced cytotoxic efficacy driven by CAR-mediated targeting. In contrast, both NK92 and CAR-NK92 exosomes showed relatively high and comparable IC_50_ values on the Her2-negative MCF-7 cells (45.894 µg/mL and 43.215 µg/mL, respectively).

## 3. Discussion

This study presents a workflow for generating CAR-NK cells using non-viral transfection, investigates the cytotoxic potential of the genetically modified cells against the widely used tumor-associated antigen Her2 and characterizes the exosomes released by the effector cells.

Since NK cells express intrinsic antiviral defense mechanisms such as toll-like receptors 2, 3, 7, and 9 [35], as well as a low expression of the LDL-receptor, these cells are considered hard to transfect using lentiviruses or gamma-lentiviruses [25]. To overcome these limitations, several techniques have been suggested, such as statin supplementation of the growth medium to upregulate LDL-receptors or using other types of lentiviruses such as the baboon envelope pseudotyped lentivector (BaEV) with a higher tropism for NK cells [36]. However, the risk of viral destruction persists due to the innate antiviral defense mechanisms. Another alternative to viral-based genetic manipulation is the use of nucleofection which involves the transfer of genetic material inside the cell using electrical currents that form pores in the plasma membrane. The risk of nucleofection is attributed to cell toxicity induced by plasma membrane disruption (leading to leakage of cellular contents and influx of extracellular fluid) [37], reactive oxygen species formation [38] and the toxicity of plasmids itself, as plasmids with higher sizes and electroporation with more than one plasmid is associated with poor viability [39]. Although transfection using electroporation was proven successful, the plasmid expression is transient, leading to a temporary expression of the chimeric antigen receptor. To overcome this limitation, the piggyBac transposon system is a promising alternative, providing seamless CAR gene integration via DNA ligation rather than DNA repair using HDR or NHEJ (as in the case of CRISPR/Cas9). The CAR-PB construct encoded a third-generation CAR anti-Her2, with an scFv derived from trastuzumab, along with the co-stimulatory molecules 4-1BB and CD28, followed by CD3ζ for downstream signaling. The plasmid also encoded for the GFP protein (allowing easier identification of nucleofected cells), and the *pac* gene, which provided resistance to the antibiotic puromycin.

The puromycin toxicity curve demonstrates that puromycin’s effects on NK92 cells are both time-dependent and concentration-dependent, with prolonged exposure (7 days) resulting in almost complete cell death even at lower concentrations. For applications requiring NK92 cell selection with puromycin, these results suggest that concentrations as low as 2 μg/mL are effective for eliminating non-resistant cells within 7 days of treatment.

Our results indicate that regular NK92 cells are highly sensitive to puromycin, with complete growth inhibition by day 7 even at low concentration, while electroporated cells expressing the *pac* gene show selective resistance to puromycin at 1–2 μg/mL, allowing cell survival by day 7. The effective selection window appears to be 1–4 μg/mL puromycin, where transduced cells survive while untransduced cells are eliminated, indicating that 4 μg/mL is likely the ideal concentration range for selective pressure. At concentrations ≥ 6 μg/mL, even transduced cells show significant toxicity by day 7, suggesting the *pac* gene expression may be insufficient to neutralize higher puromycin concentrations.

The use of puromycin selection is a well-established strategy for enriching genetically modified immune cells, including NK-92 cells engineered to express chimeric antigen receptors (CARs). Consistent with our findings, previous studies have demonstrated that untransduced NK-92 cells are highly sensitive to puromycin, with complete growth inhibition and cell death occurring within one week even at low concentrations (1–2 μg/mL) [40]. In contrast, NK-92 cells transduced with a vector encoding the puromycin N-acetyltransferase (*pac*) gene exhibit robust resistance to puromycin within a defined concentration window, typically between 1 and 4 μg/mL, allowing for effective selection of transduced populations while eliminating unmodified cells. We observed selective survival and expansion of *pac*-expressing NK-92 cells at 1–4 μg/mL puromycin, while higher concentrations (≥4 μg/mL) led to significant toxicity even in transduced cells, likely due to insufficient *pac* gene expression to fully neutralize higher drug levels. Together, these findings support the use of 1–4 μg/mL puromycin as an optimal range for selective pressure in CAR-NK92 cell engineering, ensuring efficient enrichment of the modified cells.

Based on the flow cytometry data, CAR-NK92 cells successfully express GFP, suggesting that nucleofection and gene expression occurred successfully. The use of GFP as a reporter gene is a standard method for confirming successful gene transfer and expression in CAR-engineered cells [41]. The presence of G4S linker and kappa light chains documented by fluorescence emission of anti-G4S and anti-kappa light chain antibodies confirm the presence and assembly of the CAR molecule. These results indicate successful genetic modification and expression of the designed CAR construct in NK92 cells. Several studies have established that detection of scFv components-such as the kappa light chain and the (G_4_S)_3_ linker-serves as a reliable approach for confirming CAR expression on engineered immune cells. Sarikonda et al. suggest that both protein L and anti-kappa light chain antibodies can identify CAR-expressing T and NK cells when the CAR scFv is derived from an antibody with a kappa light chain [42]. Schindler et al. showed that antibodies targeting the G4S linker can be used to detect CAR molecules, providing a specific method for monitoring CAR expression [43].

XCelligence real time cell analysis studies demonstrate that CAR-NK92 cells exhibit enhanced and sustained cytotoxicity against SK-BR3 cells, dependent on the effector-to-target ratio and mediated by their Her2-targeting CAR. This targeted cytotoxicity overcomes potential resistance mechanisms seen with untransduced NK92 cells, whose initial E:T-dependent suppression of SK-BR3 growth is temporary. While both NK92 WT and CAR-NK92 cells show some cytotoxic effects against MCF-7 cells, the CAR-NK92 cells display a more pronounced and E:T-dependent cellular death over time against SK-BR3 cells.

Western blot analysis demonstrated that exosomes isolated from both NK92 and CAR-NK92 cells robustly express canonical exosomal markers such as CD63 and Alix, confirming the identity and purity of the isolated vesicles and indicating that both wild-type and genetically modified NK92 cells produce exosomes via the same pathways [44,45]. CD3ζ molecule was identified in both Exo-NK92 and Exo-CARNK92, highlighting the role of CD3ζ as a signal transducing molecule—in Exo-NK92 in association with CD16 [46] and in Exo-CARNK92—both associated with CD16 and in the structure of CAR. Notably, a key distinguishing feature of CAR-NK92 cell-derived exosomes was the presence of the G4S linker, a unique sequence within the single-chain variable fragment (scFv) of the CAR construct. This finding provides direct evidence that the CAR molecule is incorporated into exosomes released by CAR-NK92 cells. Similar observations have been reported in recent studies, where CAR components have been detected in exosomes from engineered T or NK cells, suggesting that exosome-mediated delivery of CAR proteins may represent an additional mechanism for antigen-specific targeting [47,48].

Both exosomes derived from NK92 cells and CAR-NK92 cells express granzyme B, suggestive for a potential cytotoxic effect against tumor cells. Although not demonstrated on Exo-CAR-NK cells, Yang et al. have identified granzyme A, B, perforins and FasL as mediator for specific exo-CAR-T-mediated killing of Her2 and EGFR-positive tumor cells [49].

The presence of CAR molecules in exosomes expands the potential functional repertoire of CAR-NK92 therapy. CAR-bearing exosomes could contribute to anti-tumor effects by binding and potentially neutralizing Her2-positive target cells independently of the parental cell, and may also modulate the tumor microenvironment or facilitate intercellular communication. To further address the stability, conformation and localization of CAR molecules in exosomes, InCell ELISA has shown that CAR-NK92-Exo cells exhibit a significantly enhanced binding affinity for Her2-positive SK-BR3 cells.

Exosomal surface proteome analysis revealed that both NK and CAR-NK-derived extracellular vesicles express CD63, CD81, CD56, CD29, CD44, and CD2 on their surface. This expression repertoire was consistent with other studies that evaluated NK-derived exosomes [50,51,52,53,54]. CD56 expression reflects the NK cell line’s exosomal origin and the parental cells’ activation status.

CD25 expression was upregulated in both NK-Exo populations. It was shown in other studies that exo-NK upregulate the IL-2R on effector cells, leading to enhanced activation, proliferation, cytokine release and cytotoxicity. Hence, we suggest that besides exosomal cargo (miR-181a-3p) that upregulates IL-2 receptor expression [55], there may be a vesicular-plasmallemal fusion that might enhance the cell surface proteome with IL-2 receptors.

CD44 expression may improve exosomal adhesion to hyaluronan present in the tumoral stroma, facilitating exosome penetration and distribution in the tumor microenvironment. Although no studies described the role of CD44 in the immune cell-derived exosomes, it is known that CD44 expresion in tumor-derived exosomes facilitate tumor migration and invasion [56], CD44 expression on NK/CAR-NK-derived exosomes might improve immune cell infiltration in the tumor microenvironment.

Although only documented in MSC-derived exosomes, the presence of CD29 on exosomal surface may facilitate vesicular uptake via the CD29/CD81 interaction [57].

CD2 augments NK and T cell activation and cytotoxicity by interacting with ligands such as CD58 present on the NK and T cell surfaces and CD15 on antigen-presenting cells [58].

Our InCell ELISA results reveal that both Her2-negative MCF-7 and Her2-positive SK-BR3 breast cancer cell lines exhibit comparable baseline expression of CD63, a canonical exosomal marker indicative of active exosomal biogenesis. This suggests that both cell types maintain similar capacities for exosome production and uptake under basal conditions. NK92-derived exosomes bound to both cell lines, in SK-BR3 reaching statistical significance, consistent with non-specific or baseline exosome-cell interactions. When incubated with CAR-NK92-derived exosomes, a significant increase in optical density was observed in the SK-BR3 group, but without significant change in the MCF-7-treated cells. Comparative analysis of CAR vs. wild-type exosomes on SK-BR3 cells revealed a significant increase in absorbance at 450 nm, while in MCF-7 cells, the effect was modest, suggesting that CAR incorporation provides enhanced selectivity against target antigen-bearing cells.

These findings align with emerging evidence that CAR-engineered immune cells secrete exosomes bearing functional CAR molecules capable of targeting tumor-associated antigens [49,59]. The preferential interaction of CAR-NK92-derived exosomes with Her2-expressing tumor cells highlights their potential as a cell-free therapeutic modality that can complement or extend the cytotoxic activity of CAR-NK cells. CAR-NK cells and Exo-CAR-NK might be utilized individually or together at various stages of the tumorigenesis process.

**Figure 12:**
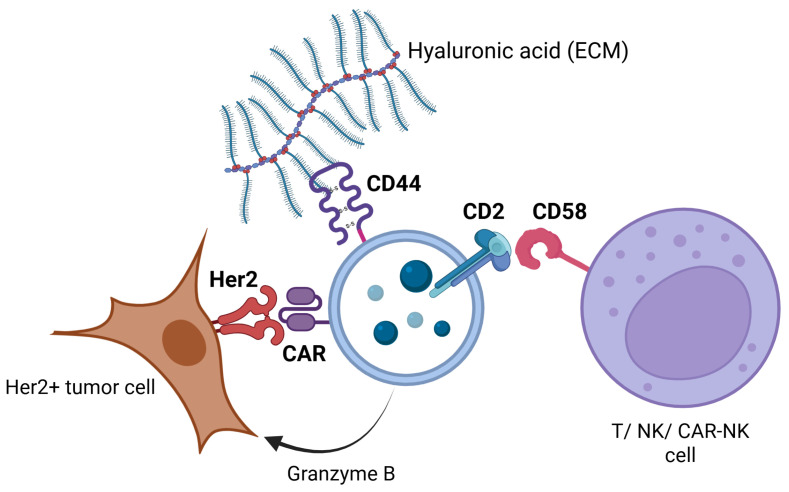
Schematic representation of the proposed mechanism by which exosomes derived from CAR-NK92 cells infiltrate the tumor microenvironment and mediate Her2-targeted cytotoxicity. Exo-CAR-NK exosomes specifically recognize and bind Her2 receptors on tumor cells, triggering downstream signaling pathways that induce tumor cell apoptosis either through direct Her2 receptor blockade or granzyme B release. The exosomal surface expression of CD44 facilitates binding to hyaluronic acid in the extracellular matrix, promoting enhanced infiltration into the tumor microenvironment. Additionally, CD2 on exosomes interacts with CD58 on T cells, NK cells, and CAR-NK cells, thereby amplifying the local immune response within the tumor microenvironment. Created in BioRender software version 4. Tirziu, A. (2025) https://BioRender.com/a1miim7 (accessed on 15 June 2025).

This targeted binding may facilitate selective delivery of cytotoxic proteins, microRNAs, or other immunomodulatory cargo contained within the exosomes, thereby amplifying anti-tumor efficacy while sparing Her2-negative cells. Moreover, exosome-mediated delivery could overcome some limitations of cellular therapies, such as poor tumor infiltration or immune evasion (Figure 12). 

Lastly, we evaluated the cytotoxic effects of exosomes derived from NK92 cells and CAR-engineered NK92 cells (exo-NK and exo-CAR-NK) on two breast cancer cell lines, SK-BR3 and MCF-7, using complementary assays to assess cell death and viability. Our results demonstrated that while both exosome types induced cell death as indicated by alamarBlue viability assays, a significant reduction in SK-BR3 cell viability was observed at higher doses of exo-CAR-NK.

The observed selective efficacy of exo-CAR-NK against SK-BR3 cells is consistent with the specific targeting of Her2, a receptor overexpressed in SK-BR3 cells but absent or expressed at low levels in MCF-7 cells [60]. CAR engineering of NK cells to express a receptor targeting Her2 enhances the cytotoxic activity of their exosomes, likely through surface presentation of CAR or the delivery of cytotoxic molecules, leading to higher rates of tumor cell death [61,62]. This supports the growing evidence that engineered exosomes can recapitulate the targeting capabilities of their parental immune cells and serve as potent cell-free therapeutic agents with improved specificity [63].

The relatively reduced effects of both exosome types on MCF-7 cell viability and metabolic activity suggests the importance of antigen-specific interactions in mediating exosome-induced cytotoxicity. Previous studies have highlighted that NK cell-derived exosomes can exert cytotoxicity through ligand-receptor interactions, including the engagement of activating receptors and delivery of effector molecules such as perforin, granzyme B, and Fas ligand [64]. However, in the absence of target antigen recognition, as with the MCF-7 model, these effects appear relatively lower, aligning with our findings.

Our results are in agreement with prior reports showing that CAR-expressing immune effectors display enhanced cytotoxicity against antigen-expressing tumor targets [65]. Additionally, the dose-dependent decrease in SK-BR3 viability induced by exo-CAR-NK suggests a scalable therapeutic potential whereby exosomal protein concentration can be optimized for maximal antitumor efficacy.

There are limitations to our study that should be considered. While alamarBlue assays provide complementary insights into cell death and metabolic activity, other mechanisms of exosome-mediated cytotoxicity, such as induction of apoptosis or immune modulation, remain to be elucidated. Furthermore, in vivo studies are required to confirm the therapeutic feasibility and safety profile of these engineered exosomes.

## 4. Materials and Methods

**Figure 13:**
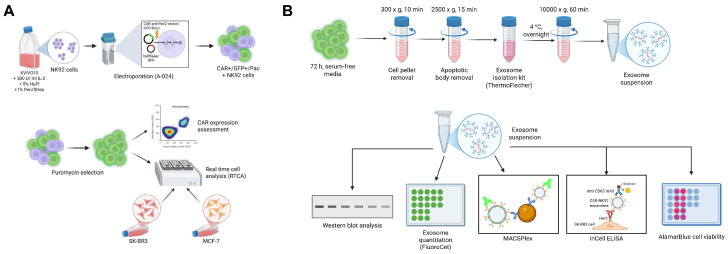
Schematic workflow for this study. (**A**) NK92 cells were cultured in XVIVO10 medium supplemented with 5% human plasma, 1% Pen/Strep and 500 IU/mL IL-2. NK92 cells were electroporated using the A-024 Amaxa Nucleofector II program. Puromycin selection was performed using various concentrations to determine the optimal concentration. CAR expression assessment was performed using flow cytometry and cytotoxicity assays were performed using SK-BR3 or MCF-7 cells at various E:T ratios in RTCA experiments. (**B**) CAR-NK92 cells and NK92 WT cells were incubated for 72 h in serum-free media to deplete the cell culture from human plasma-derived exosomes and to stimulate NK92-specific exosome release. Exosomes were isolated using serial centrifugations and precipitation with Exosome Isolation Reagent (Thermo Fischer). Exosome quantification was assessed using the FluoroCet fluorimetric assay and their protein content was assessed using the NanoDrop spectrophotometer. Surface protein and cargo was analyzed using Western blot and MACSPlex. InCellELISA was performed on SK-BR3 and MCF-7 cells treated with exosomes to evaluate the specific exosomal binding capacity to Her2. Exosomal cytotoxic potential was investigated using alamarBlue viability assay by incubating SK-BR3 and MCF-7 cells with exosomes from NK92 and CAR-NK92 cells at different concentrations expressed as µg of exosomal protein.

The schematic workflow for this study is summarized in Figure 13.

### 4.1. Cell Cultures

The NK-92 cell line was purchased from ATCC and further expanded in the XVIVO-10 medium, supplemented with 5% human plasma (American Type Culture Collection, cat. no. CRL-2407, Manassas, VA, USA), 500 IU/mL IL-2 (StemCell Technologies, cat. #78145, Vancouver, BC, Canada) and 1% Penicillin/Streptomycin (Pen/Strep; Sigma Aldrich, Saint Louis, MO, USA; cat. #P4333), at a cellular density of 4–5 × 10^5^ cells/mL. 

The Her2+ SK-BR3 cell line (ATCC HTB-30^™^) was plated in culture flasks containing the Modified McCoy’s 5a (Gibco BRL, Invitrogen, Carlsbad, CA, USA) medium supplemented with 10% fetal calf serum (FCS, PromoCell, Heidelberg, Germany) and 1% Pen/Strep solution, at a cell culture density of 3–6 × 10^5^ cells/cm^2^. 

The MCF-7 Her2-, ER+, PR+ cell line was expanded in Dulbecco’s Modified Eagle Medium (DMEM, Sigma-Aldrich, Saint Louis, MO, USA; cat. #D0822), supplemented with 10% FCS and 1% Pen/Strep at a cell culture density of 3 × 10^4^ cells/cm^2^. 

All cellular types were grown at 37 °C in a humid atmosphere containing 5% CO_2_. The medium was replaced every 2–3 days until reaching 80–90% confluence (for adherent cells). The adherent cells were detached from the culture flasks using a 0.25% Trypsin-EDTA solution (Sigma-Aldrich, cat. #T4049). 

For suspension cells, after centrifugation for 10 min at 300× *g*, the cells were re-plated in suitable culture flasks at a density of 2–5 × 10^5^ cells/cm^2^.

### 4.2. Flow Cytometry Assessment of the Target Cells

Her2 expression in target cells was assessed using flow cytometry. SK-BR3 and MCF-7 cells were detached from culture flasks using 0.25% Trypsin-EDTA. After two washing steps with 1x phosphate-buffered saline (PBS; Thermo Fischer Scientific, Waltham, MA, USA, cat. #J61196.AP), the cells were resuspended in 100 µL of 1x PBS at a concentration of 10^5^ cells/mL, followed by an incubation in the dark at room temperature for 30 min with a PE-conjugated mouse anti-human Her-2/neu antibody (clone NEU 24.7; BD Biosciences, San Jose, CA, USA, cat. #340552) antibody (following the manufacturer’s specified dilution protocol). Cells were then washed twice with 1 mL Cell Wash Solution (BD Biosciences, San Jose, CA, USA) each and resuspended in 500 μL of the same solution for further analysis on a BD FACSVerse flow cytometer. Acquisition and data analyses were performed using BD FACSuite (BD BioSciences, version 5.1).

### 4.3. CAR Anti-Her2 Vector Design

**Figure 14:**
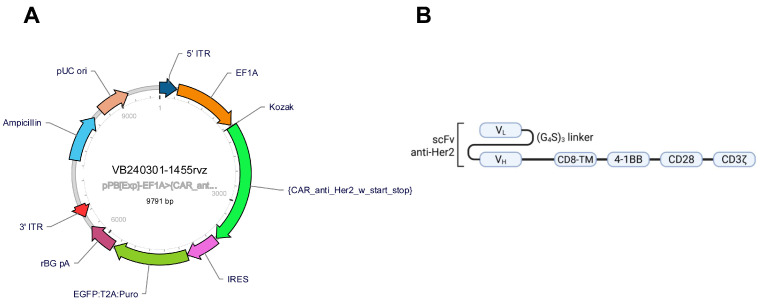
Design and Structure of the Third-Generation Anti-Her2 CAR Vector. (**A**) Schematic representation of the pPB [Exp]-EF1A>CAR construct generated using the VectorBuilder platform. This bi-cistronic piggyBac transposon-based plasmid (9791 bp) contains the anti-Her2 CAR sequence driven by the EF1A promoter and flanked by 5′ and 3′ inverse terminal repeats (ITRs). The vector includes a Kozak consensus sequence, a CD8a leader, and the CAR transgene, followed by an internal ribosome entry site (IRES) and a dual selection cassette expressing EGFP and a puromycin resistance gene (*pac*) linked via a T2A sequence for bicistronic expression. This design facilitates both visual and antibiotic selection of successfully transduced cells. (**B**) Structural diagram of the anti-Her2 chimeric antigen receptor (CAR). The extracellular domain consists of a single-chain variable fragment (scFv) derived from trastuzumab (anti-Her2), composed of VH and VL regions linked by a flexible (G_4_S)_3_ linker. This is fused to a CD8a hinge and transmembrane domain. The intracellular signaling domain contains the co-stimulatory molecules 4-1BB and CD28, followed by the CD3ζ activation domain.

The third-generation chimeric antigen receptor used in this study comprises an extracellular domain that interacts with the Her2 receptor via the VH and VL chains that are linked with a G4S linker. The CD8a transmembrane domain is linked to the extracellular domain via a CD8a hinge sequence. The intracellular domain contains the co-stimulatory molecules CD28 and 4-1BB, while the signal transduction pathway is activated by the CD3ζ molecule. To improve clone selection, along with the CAR sequence, a puromycin-resistance gene (*pac*) was added to the construct. Recognition of successful transduction is mediated by the simultaneous expression of GFP (Figure 14).

The CAR anti-Her2 vector designed using the VectorBuilder platform is a bicistronic plasmid that includes between the 5′ and 3′ inverse tandem repeat elements (ITRs) the CAR sequence and the dual selection marker EGFP-Puromycin resistant transgene. The CAR DNA sequence comprises the EF-1a promoter, a Kozak restriction sequence, a CD8a leader sequence, the Trastuzumab scFv VH and VL, joined by a (G_4_S)_3_ linker, a CD8a hinge region, a CD8a transmembrane domain, CD28, 4-1BB, and CD3-ζ. The Trastuzumab VH and VL were obtained by reverse translating the amino acid FASTA sequence (retrieved from PDB, id 8Q6J) into DNA using EMBL BackTranseq, followed by codon optimization using Gensmart (GenScript) [66].

### 4.4. NK-92 Cell Transfection

NK92 cells in the log phase were seeded at a density of 2.5 × 10^6^ cells/well in culture media for 24 h. Cells were centrifuged at 100× *g*, 10 min and resuspended in 100 µL Nucleofector Solution (Lonza Biosciences, cat. #VPA-1005, Basel, Switzerland), along with the plasmid vectors. Two vectors were used for nucleofection—one vector encoding the hyPBase transposase and the fluorescent reporter mCherry (VectorBuilder) and the CAR-encoding vector in a 1:1 ratio (2 ng DNA/plasmid). Cell suspensions were transferred in nucleofection cuvettes (Lonza Biosciences, Basel, Switzerland) and electroporated using the A-024 program on the Amaxa Nucleofector II System (Lonza Biosciences, Walkersville, MD, USA). Immediately after electroporation, the cells were resuspended in a 6 well-plate containing fresh culture medium, at 37 °C and incubated for 72 h, 5% CO_2_. pMaxGFP was used as a positive control, while cells electroporated without DNA (w/o DNA) were used as negative control.

### 4.5. Puromycin Killing Curve

In a 24-well plate, 5 × 10^4^ NK-92 cells/well were seeded in their complete growth media. Various concentrations of Puromycin dihydrochloride (Thermo Fischer, cat. #A1113803)—0, 1, 2, 4, 6, 8, 10 µg/mL were added to the media. To force the selection process, the puromycin-containing media was replaced every 2–3 days. The optimal dose for puromycin was determined by the concentration of the antibiotic that induces 95% cellular death in approximately 7 days. The optimal dose was further used for selecting the CAR-positive NK-92 cells. 

### 4.6. Transfection Efficiency and Cellular Viability Assessment

At 72 h post-transfection the NK-92 cells were assessed by flow cytometry based on the GFP-expression levels, along with the CAR anti-Her2 expression. CAR expression was evaluated using the Rabbit anti-Human G4S linker (Cell Signaling Technology, cat. #71645, Leiden, The Netherlands) and goat anti-rabbit AlexaFluor 594 (Thermo Fischer Scientific, cat. #A-11012), diluted according to the manufacturer’s instructions. The presence of kappa light chains in the CAR structure was evaluated using the mouse anti-human kappa light chain monoclonal antibody (SinoBiological, cat. #IHC043), along with the AlexaFluor 647 conjugated donkey anti-mouse secondary antibody (Thermo Fischer Scientific, cat. #A-31571, Carlsbad, CA, USA). The GFP+/PerCP+/APC+ cells were considered effectively transduced. 

### 4.7. In Vitro Cytotoxicity Assay

To examine the cytolysis of adherent target cells by transduced cells over time, Her2+ positive SK-BR3 and MCF7 cells were seeded on 16-well E-plates 16 PET (Agilent Technologies, Santa Clara, CA, USA, cat. #30-060-0890) at a density of 5000 cells/well and cultured for 24 h before co-culturing them with CAR-expressing cells and untransduced cells at effector-to-target ratios of 1:1, 5:1 and 10:1, respectively. The effector-mediated target cell death was monitored in real-time using the xCELLigence system (Agilent Technologies, Santa Clara, CA, USA), which measures the cell index (CI) proportional to the cellular impedance. RTCA provides real-time data regarding cell number, size, and adherence based on the CI dynamics. The viability of target cells, indicated by the CI, was monitored for 72 h. Target cells alone were used as a negative control.

### 4.8. Exosome Isolation

The NK92 cells, along with CAR-NK92 anti-Her2 cells were cultured for 72 h in serum free-media to increase exosome production. Cells were further centrifuged at 300× *g*, 10 min, and their supernatant was collected. The supernatant was centrifuged at 2500× *g*, 15 min to remove cell debris and apoptotic bodies, and incubated with Total Exosome Isolation Reagent (from cell culture media) (ThermoFischer Scientific, cat. #4478359, Carlsbad, CA, USA) in a ratio of 2:1 overnight, at 4 °C. In the following day, the tubes were centrifuged at 10,000× *g*, 1 h, 4 °C and the sediment was resuspended in 100 µL Exosome Resuspension Buffer (ThermoFischer Scientific, cat #4478545, Carlsbad, CA, USA). The total exosomal protein content in the samples was assessed using the NanoDrop ND1000 Spectrophotometer (ThermoFischer Scientific, cat. #15596026, Wilmington, DE, USA) based on the absorbance at 230 nm. 

Exosome quantification was performed using the FluoroCet kit (System Biosciences, cat. #FCET96 A-1, Palo Alto, CA, USA). Exosomal suspensions were lysed using Exosome Lysis Buffer on ice for 30 min. The exosomal lysate was incubated for 20 min in reagent A (with detection reagent, 1:100) and reagent B (containing acetylcholine chlorine) in a 1:1:1 ratio in a dark room, at room temperature. The standard fluorescence curve was generated using serial dilutions of the FluoroCet standard (1:64 in Reaction Buffer). The results are expressed as optical densities at an excitation spectrum of 530–570 nm and emission spectrum of 595 nm.

### 4.9. Identification of Exosomal Markers and the Presence of CAR on Exosomes

Exosomes were suspended in Laemli’s Sample Buffer and incubated for 5 min at 95 °C. The exosomal lysates were migrated on a 10% polyacrylamide gel at 100 V, 90 min and transferred on an Amersham™ 0.2 µm nitrocellulose membrane (GE Healthcare Life Sciences, cat. #1060001, Pittsburgh, PA, USA) using the Hoeffer TE62 Standard Transfer Tank, at 150 mA, 90 min (Hoeffer, Bridgewater, MA, USA). The nitrocellulose membranes were marked with exosome-specific antibodies (rabbit anti-CD63 primary antibody, cat. #25682-1-AP, rabbit anti-Alix, cat. #12422-1-AP, ProteinTech, Martinsried, Germany), anti-granzyme B antibodies (rabbit anti-granzyme B, cat. #13588-1-AP, ProteinTech, Martinsried, Germany) and CAR-specific antibodies (rabbit anti-CD3ζ, cat. #88083; rabbit anti-G4S linker, cat. #71645, Cell Signaling Technology, Leiden, The Netherlands), respectively, overnight, at 4 °C. The membrane was washed with Tris-buffered saline with Tween 20 (TBS-T), 0.1% and incubated for 2 h with anti-mouse (cytiva, car. #NA931V, Marlborough, MA, USA) and anti-rabbit HRP-conjugated antibodies (cytiva, cat. #NA934V, Marlborough, MA, USA). After the second wash step, the membranes were incubated for 5 min in SignalBright Pro ECL solution (ProteinTech, cat. #PK10011, Martinsried, Germany) and analyzed using the iBright FL1000 (Thermo Fisher Scientific) at 1 min exposure time. 

Exosomal surface proteome was assessed using the MACSPlex EV IO Detection Kit (Miltenyi Biotec, cat. #130-108-813, Bergisch Gladbach, Germany) by incubating the exosomal suspension with 15 µL of exosome capture beads along with APC-conjugated exosome detection antibodies (anti-CD63) for 1 h. After washing the exosomal suspension, the analysis was performed on the BD FACSVerse flow cytometer by assessing the fluorescence emitted on FITC/PE/APC channels. The results are presented as median fluorescence intensities ± SEM after performing background subtraction of unbound exosomal capture beads.

### 4.10. Her2-Specific Exosomal Binding Assessment

To evaluate the capacity of Exo-CARNK92 to bind to Her2+ cells, a modified InCell ELISA (Thermo Fischer Scientific, cat. #62200, Carlsbad, CA, USA) protocol was performed. 5000 SK-BR3 and MCF-7 cells/well were cultured for 24 h on a 96-well plate. On the following day, the cells were fixated with methanol, 4% and permeabilized using SurfactAmps X100 and hydrogen peroxide, 1% in TBS-T. The wells were further blocked with Blocking Buffer and incubated for 24 h with 10 µg of exosomes from NK-92 WT cells and CAR-NK92 cells, respectively. After washing the wells, anti-CD63 mouse antibodies (1:50) were added to the cells and incubated for an additional 24 h. HRP conjugate was added after washing the plate with SurfactAmps20. TMB substrate was added into the wells and after 15 min, the reaction was stopped using Stop Solution. The plate was analyzed using the VarioScan plate reader (Thermo Fischer Scientific, cat. #VA000010C) at 20 min after the addition of Stop Solution. The results are displayed as the mean optical densities at 450 nm measured from 12 points of each well.

### 4.11. Exosomal Cytotoxic Effect Assessment

SK-BR3 and MCF-7 cells were seeded for 24 h at 5000 cells/well in a 96-well plate (Microtest Primaria™, cat. #353872, BD Biosciences, San Jose, CA, USA) and incubated for 24 h at 37 °C, 5% CO_2_. Exosomes from NK92 cells and CAR-NK92 cells were added in the corresponding wells in various quantities (1, 5, 10 µg of exosomal protein). After 24 h of incubation, 10% of the total well volume of resazurin (alamar Blue™ Cell Viability Reagent, Thermo Fischer Scientific, Carlsbad, CA, cat. #DAL1025) and analyzed at 4–10 h using the Tecan Infinite M200 Pro plate analyser (Tecan, Grödig, Austria) at *λ* = 570 nm and 600 nm, respectively. The cell viability is assessed on the percentage of reduced resazurin that results from the metabolically active cells:

$$\%AB\;reduction=\;\frac{{(\varepsilon }_{oxidized,600nm}\;\times \;A_{570nm}\;)-\;{(\varepsilon }_{oxidized,570nm}\;\times \;A_{600nm}\;)}{{(\varepsilon }_{reduced,570nm}\;\times \;N_{570nm}\;)-\;{(\varepsilon }_{reduced,600nm}\;\times \;N_{600nm}\;)}\times 100$$

where

$\varepsilon_{oxidized,600nm}$—oxidized alamarBlue molar extinction coefficient at 600 nm;

$A_{570nm}$—cell absorbance at 570 nm;

$\varepsilon_{oxidized,570nm}$—oxidized alamarBlue molar extinction coefficient at 570 nm;

$A_{600nm}$—cell absorbance at 600 nm;

$\varepsilon_{reduced,570nm}$—reduced alamarBlue molar extinction coefficient at 570 nm;

$N_{570nm}$—negative control (culture media without cells) absorbance at 570 nm;

$\varepsilon_{reduced,600nm}$—reduced alamarBlue molar extinction coefficient at 600 nm;

$N_{600nm}$—negative control (culture media without cells) absorbance at 600 nm.

### 4.12. Statistical Analysis

The data were presented as the mean ± standard error of mean (SEM). All experiments were performed in triplicate to ensure reproducibility and statistical reliability. Normally distributed data were analyzed using Student’s paired *t*-test and one-way ANOVA. A value of *p* < 0.05 was considered statistically significant. All analysis was conducted using GraphPad Prism version 8.0.2 software. Western blot signal intensity was analyzed using ImageJ version 1.54g.

## 5. Conclusions

In this study, we successfully generated and characterized anti-Her2 CAR-NK92 cells demonstrating their potent and selective cytotoxicity against Her2-positive cancer cells. Furthermore, exosomes from CAR-NK92 cells were isolated and characterized. Using the piggyBac transposon system and puromycin selection, we achieved highly efficient CAR expression in NK92 cells, with robust Her2-specific cytotoxic activity confirmed in vitro. Notably, exosomes derived from CAR-NK92 cells retained CAR molecules and selectively targeted Her2-positive cells. Our findings highlight the potential of both CAR-NK92 cells and their exosomes as innovative and versatile immunotherapeutic agents for the treatment of Her2-positive tumor cells. The use of exosomes may further overcome some limitations of cell-based therapies, offering a promising cell-free platform for targeted cancer immunotherapy. Future studies will be necessary to evaluate the in vivo efficacy, safety, and scalability of this approach, as well as to explore its applicability to other tumor-associated antigens.

## Data Availability

Data are available upon reasonable request to the submitting author.

## Abbreviations

The following abbreviations are used in this manuscript:

aR^2^	Adjusted Coefficient of Determination
CAR	Chimeric Antigen Receptor
CAR-NK	Chimeric Antigen Receptor-Engineered Natural Killer (cell)
CD3ζ	CD3 zeta chain (T-cell co-receptor signaling molecule)
CRISPR	Clustered Regularly Interspaced Short Palindromic Repeats
D0, D3, D7	Day 0, Day 3, Day 7 (timepoints)
DNA	Deoxyribonucleic Acid
df	Degrees of Freedom
E:T	Effector-to-Target (cell ratio)
(e)GFP	(Enhanced) Green Fluorescent Protein
ELISA	Enzyme-Linked Immunosorbent Assay
FACS	Fluorescence-Activated Cell Sorting
FBS	Fetal Bovine Serum
FSC	Forward Scatter (Flow Cytometry)
F	F-statistic (analysis of variance)
GVHD	Graft-versus-Host Disease
Her2	Human Epidermal Growth Factor Receptor 2
hyPBase	Hyperactive PiggyBac Transposase
ICOS	Inducible T-cell COStimulator
ILV	Intraluminal Vesicle
ITR	Inverted Terminal Repeat
mAb	Monoclonal Antibody
mRNA	Messenger RNA
miRNA	MicroRNA
MVB	Multivesicular Body
NK	Natural Killer (cell)
NK92	NK92 cell line (human natural killer cell line)
ns	Not Significant
OD	Optical Density
PB	PiggyBac (transposon system)
*p*	*p*-value (probability value in statistical tests)
RAU	Relative Absorbance Units
RTCA	Real-Time Cell Analysis
scFv	Single-chain Variable Fragment
SD	Standard Deviation
SE	Standard Error
SEM	Standard Error of the Mean
SNARE	Soluble NSF Attachment Protein Receptor
SSE	Sum of Squared Errors
TCR	T Cell Receptor
WT	Wild-Type
VSV-G	Vesicular Stomatitis Virus Glycoprotein (used in viral pseudotyping)
LDL	Low-Density Lipoprotein
